# Effects of calcination temperature for rate capability of triple-shelled ZnFe_2_O_4_ hollow microspheres for lithium ion battery anodes

**DOI:** 10.1038/srep46378

**Published:** 2017-04-18

**Authors:** Hojin Hwang, Haeun Shin, Wan-Jin Lee

**Affiliations:** 1 Chonnam National University, School of Chemical Engineering, Gwangju, 61186, Republic of Korea

## Abstract

Triple-shelled ZnFe_2_O_4_ hollow microspheres (ZFO) as anode materials for lithium ion battery are prepared through a one-pot hydrothermal reaction using the composite solution consisting of sucrose in water and metal ions in ethylene glycol (EG), followed by different calcination processes. The architectures of ZFO micro spheres are differently synthesized through a mutual cooperation of inward and outward ripening with three different calcination temperatures. Thin triple-shelled ZnFe_2_O_4_ hollow microspheres calcined at 450 °C (ZFO-450) delivers a high reversible capacity of 932 mA h g^−1^ at a current density of 2 A g^−1^ even at the 200^th^ cycle without obvious decay. Furthermore, ZFO-450 delivers 1235, 1005, 865, 834, and 845 mA h g^−1^ at high current densities of 0.5, 2, 5, 10, and 20 A g^−1^ after 100 cycles. Thin triple-shelled hollow microsphere prepared at an optimum calcination temperature provides exceptional rate capability and outstanding rate retention due to (i) the formation of nanoparticles leading to thin shell with morphological integrity, (ii) the facile mass transfer by thin shell with mesoporous structure, and (iii) the void space with macroporous structure alleviating volume change occurring during cycling.

The architectural features for ternary transition metal oxides (TTMOs, TM = Zn, Fe, Co, Mn, Ni, and Cu) such as zinc ferrite (ZnFe_2_O_4_) with a high theoretical capacity of 1000 mA h g^−1^ to replace carbonaceous materials such as graphite (<372 mA h g^−1^) in application of various portable devices, electric vehicles, and lithium ion batteries (LIBs) are directly related to the improvement of electrochemical performance with excellent rate capability and exceptional cycling stability^1^,^2^,^3^,^4^. Such morphologies originate from the design of mesoporous materials with their high specific surface area, high mesopore, and wide average pore distribution^5^,^6^. More specifically, the dependence on calcination temperature as post-temperature treatment acts as an indispensable factor in designing porous structures owning to its crucial influence on the formation of pores. To cope with these issues, multi-shelled hollow metal oxide microspheres as anode material of LIBs have great attention because of their morphologies with appropriate shell and void enhance lead to the enhancement of electrochemical performances by maintaining structural integrity suitable for severe volume expansion^7^,^8^,^9^.

The architectures with multi-shelled hollow morphologies are commonly synthesized through template-mediated synthesis, inward and outward ripening, and Kirkendall effects^10^,^11^,^12^,^13^,^14^,^15^. Among template-mediated routes, a one-pot hydrothermal method makes it easier to react at a time the composite solution of sucrose and metal ions in water and ethylene glycol (EG) at proper temperature, and subsequently the decomposition of core carbon. The EG generates zinc/iron-glycolate (ZnFe-glycolate) through the process of oligomerization, and then ZnFe-glycolate gets well-dispersed into sucrose-based carbon consisting of ZnFe-glycolate/carbon microspheres via the polyol reaction of EG^16^. This approach is a tailored-architecture way accessible to synthesize multi-shelled hollow microspheres composed of nanostructured metal oxide by chemically induced self-assembly in a one-pot hydrothermal environment^17^,^18^,^19^,^20^. The aqueous solution of metal precursor in this process is completely mixed with the aqueous solution of sucrose, followed by hydrothermal reaction at a proper temperature. This hydrothermal reaction generates a spherical metal oxide-carbon composite by executing the condensation polymerization and carbonation of sucrose and the formation of metal-glycolate at the same time, and subsequently the hollow metal oxide microspheres are formed by removal of carbon via high temperature calcination^21^,^22^. Recently, the preparations of hollow structured metal oxides by a one-pot hydrothermal reaction have been studied because of their structural advantages such as easy control of morphology and facile monodisperse size distribution for the enhanced electrochemical performance with rate capability by depressing volume expansion^3^,^23^,^24^,^25^.

Among the metal oxides hollow spheres, the multiple-shelled ZnFe_2_O_4_ hollow microspheres are differently prepared with different calcination temperatures, easily predicting a strong effect of electrochemical performances. To date, the optimum design of multiple-shelled ZnFe_2_O_4_ hollow spheres with various calcination temperatures is rarely reported. The multiple-shelled ZnFe_2_O_4_ hollow spheres using a one-pot hydrothermal reaction under different calcination temperatures are fabricated through the mutual cooperation of inward and outward ripening, which consist of both mesoporous shells and macroporous voids influencing on the improvement of electrochemical performance^26^,^27^,^28^. Inward ripening begins to initiate mass rearrangement from the outer surface, whereas outward ripening starts mass transfer from the center of a crystallite aggregate. Particularly, thin multiple-shelled ZnFe_2_O_4_ hollow spheres synthesized at an appropriate calcination temperature are suitable for enhancing specific capacity and rate capability, and cycling stability due to the following reasons. Firstly, the nanoparticles comprising of mesoporous structured shells offer the structural stability accommodating volume expansion during the process of Li^+^ insertion and extraction, and the sufficient ability of lithium storage owning to their formation of abundant electrochemical sites^29^,^30^. Secondly, the interparticle mesoporous spaces within thin shells makes it easier to penetrate the electrolyte owning to surface permeability. The interparticle mesoporous spaces reduce the diffusion length of Li^+^, facilitate mass transfer by creating the numerous interfacial regions within shells, and hold the capacity of improving the electrochemical kinetics. Thirdly, the wide void with macroporous structure is deeply dependent on the alleviation of severe volume change during lithiation/delithiation^23^,^24^. The wide void also plays an important role in the reservoir of electrolyte as well as the integrity of structure. The wide void has a large influence on maintaining the capacity by maximizing electrochemical reaction^31^. This desirable architecture suitable for improving the electrochemical performance is largely governed by different calcination temperatures in preparing the multiple-shelled ZnFe_2_O_4_ hollow microspheres.

Herein, we report the first fabrication of thin triple-shelled ZnFe_2_O_4_ hollow spheres using a one-pot hydrothermal technique of a solution involved with zinc, iron ions, and sucrose leading to ZnFe-glycolate/carbon composite, followed by decomposition of core carbon at an optimum calcination temperature. The calcination temperature has a great effect on the transformation of triple-shelled ZnFe_2_O_4_ hollow structure so as to obtain excellent specific capacity and good electrochemical retention without any fading of rate capability. The tailored architecture with thin triple-shelled ZnFe_2_O_4_ hollow structure at an optimum calcination temperature provides the advantage in shortening the distance of mass transfer and sustaining the structural integrity, which leads to superior electrochemical performance delivering 932 mA h g^−1^ at a constant density of 2 A g^−1^ even at the 200^th^ cycle.

## Results

In a one-pot hydrothermal reaction of the precursor solution of ZnFe_2_O_4_ microspheres comprising of sucrose and zinc nitrate and iron nitrate in water and EG as solvents, the carbon is formed by the condensation polymerization reaction and carbonation of sucrose, and concurrent ZnFe-glycolate is formed by the polyol reaction of EG with zinc and iron ions, followed by the generation of ZnFe-glycolate/carbon microspheres. The formation of ZnFe-glycolate is described as follows^20^:


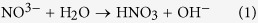



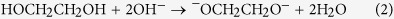



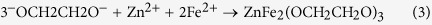


**Figure 1:**
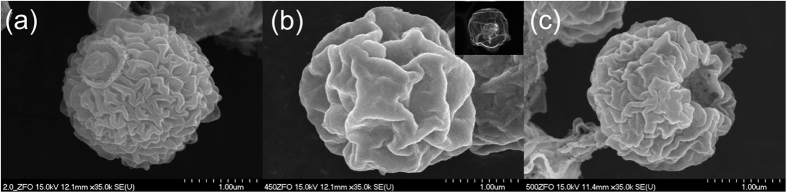
SEM images of (**a**) ZFO-400, (**b**) ZFO-450, and (**c**) ZFO-500.

The ZnFe-glycolate is well-adsorbed within the hydrophilic sucrose-based carbon, comprising ZnFe-glycolate/carbon microspheres. Afterwards, various ZnFe_2_O_4_ hollow microspheres are formed by contracting ZnFe-glycolate/carbon microspheres according to calcination process. The SEM images of ZFOs prepared at different calcination temperatures are demonstrated in Figs 1 and S1. The ZFOs are composed of the thin shell with wrinkly and rough surfaces. As for the inset image of ZFO-450 (Fig. 1), thin triple-shelled hollow microsphere is clearly witnessed, which consist of the contour of ball in ball generating wide voids and thin shells with numerous interparticle pores. The wide void with macroporous morphology and numerous interparticle pores within mesoporous thin shell governs the enhancement of electrochemical performance by encumbering the ZFO pulverization caused from the volume expansion during lithiation/delithiation.

X-ray diffraction (XRD) patterns of ZnFe-glycolate and ZFO with different calcination temperatures are shown in Figure S2. Two peaks at around 11 and 13° are characteristic of ZnFe-glycolate. The most striking feature is the disappearance of the ZnFe-glycolate peaks above 400 °C. For all of the ZFO samples, the diffraction peaks are observed at 2θ = 30.0, 35.3, 36.9, 42.9, 53.3, 56.8, 62.3, 70.7, and 73.8°, respectively, corresponding to (220), (311), (222), (400), (422), (333), (440), (620), and (533) planes. The diffraction peaks of ZFOs is identified with spinel crystalline structure (JCPDS card No. 82–1049)^32^. The diffraction peaks with the high crystallinity are stronger and sharper with the increase in crystal size, which depends on the formation of morphology by calcination temperature. The diffraction peaks of ZFO-450 are weaker and wider compared to those of ZFO-400 and ZFO-500, implying that the morphology of ZFO-450 is thin shell microsphere consisting of smaller crystalline. In contrast, the diffraction peaks of ZFO-400 and ZFO-500 are sharper than those of ZFO-450, indicating the formation of thick-shelled microsphere comprising larger crystalline.

**Figure 2:**
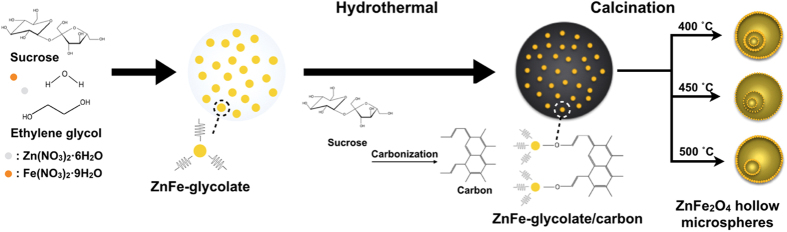
Schematic illustration for the synthesis of various shelled ZnFe_2_O_4_ hollow microspheres.

The schematic diagram for the synthesis of ZFOs is illustrated in Fig. 2. Firstly, the sucrose as a carbon precursor in water is completely mixed with zinc nitrate and iron nitrate as metal precursors in ethylene glycol, and subsequent a hydrothermal reaction at 190 °C for 24 h. In the process of reaction, the composite solution turned into light black color, depicting that ZnFe-glycolate/carbon microspheres are formed. Secondly, ZnFe-glycolate/carbon microspheres transform into ZFO with triple-shelled hollow structure by inward or outward ripening during the calcination process. More specifically, the heat transfer is progressed along the radial direction from the surface layer of microspheres. Then, the outermost layer of the ZnFe-glycolate/carbon is converted into ZFO outer shell of solid state, inducing heterogeneous contraction caused by temperature gradient during the inward ripening. The ZnFe-glycolate from inner ZnFe-glycolate/carbon is easily detached, forming the wide voids between the shells. The detached inner core generates the inner shell by occurring the outward ripening along the inner microspheres from the outer ones due to temperature gradient. Finally, the triple-shelled ZnFe_2_O_4_ hollow microspheres are formed by the inward, outward ripening, and the removal of carbon spheres. In other words, the ZFOs are formed at different calcination temperatures, each of which generates various shell architectures. The different calcination temperatures have impacts on the formation of the triple-shelled hollow morphology due to the degree of crystallization and the rate and amount of CO_2_ gas released from carbon spheres. The structural variations of each shell thickness and each void width generated with different calcination temperatures intensely influences on the electrochemical performance owning to the mechanism of the electrochemical reaction. The ZnFe_2_O_4_ hollow microspheres synthesized at an optimum calcination process lead to the improvement of rate capability and cycling stability owning to the generation of thin shell and wide void.

**Figure 3:**
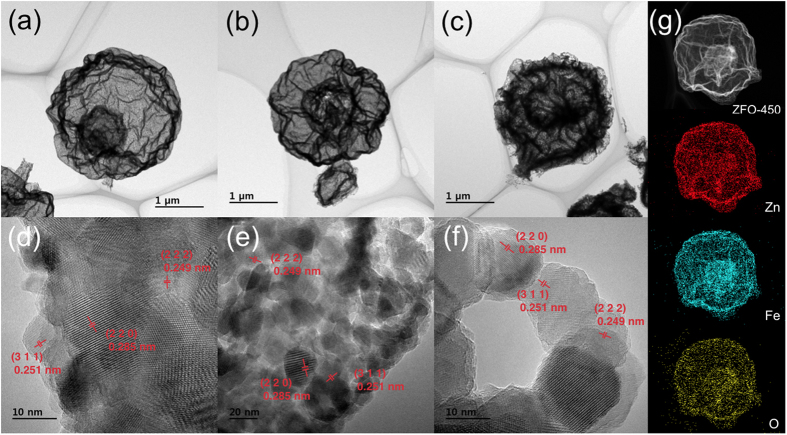
HRTEM images for (**a**,**d**) ZFO-400, (**b**,**e**) ZFO-450, and (**c**,**f**) ZFO-500 with different calcination temperatures; (**g**) STEM image and corresponding EDAX elemental mappings of Zn, Fe, and O for ZFO-450.

TEM images of the ZnFe_2_O_4_ hollow spheres calcined at 400, 450, and 500 °C in air for 4 h at a heating rate of 2 °C min^−1^ are shown in Figs 3 and S3. The ZnFe-glycolate/carbon microspheres through a one-pot hydrothermal reaction are converted into various ZnFe_2_O_4_ hollow structures with different calcination temperatures. All of the samples with the shells and inner core are composed of the well-connected round network with nanoparticles. From Fig. 3a–c, the diameter of hollow microspheres decreases with the rise in calcination temperature, representing that the diameters of ZFO-400, ZFO-450, and ZFO-500 reduce in the order of 2.51, 2.12, and 2.05 μm, respectively, due to the shrinkage of microspheres. The ZFO-450 has well-developed thin triple-shelled microspheres with macroporous void spaces, while ZFO-400 and ZFO-500 possess thick triple-shelled microspheres and thick double-shelled ones, respectively^33^. Thin triple-shelled ZnFe_2_O_4_ hollow microsphere (ZFO-450) are composed of the outer shell, void, inner shell, and inner hollow core along the radial direction from the surface. Thin shell formed by nanoparticles largely offers high specific surface areas with a lot of electrochemical active sites and short diffusion lengths facilitating mass transfer^34^. The wide void between shells leads to the facile mass transfer by storing abundant electrolyte and protects against the damage of microspheres by providing tremendous spaces hindering severe volume change during lithiation/delithiation, whereas the increase in crystal size gives rise to the decreased electrochemical sites. As a result, the formation of wide void and tiny nanoparticles leads to the best electrochemical performance. There are abundant interstitial spaces between nanoparticles comprising of shells. The nanoparticles reduce the internal stress caused by the strains between nanoparticles, which protect pulverization of active materials originated in severe volume expansion during lithiation/delithiation. Thus, the huge volume expansion incurring the capacity fade is prohibited by the structural integrity of thin triple-shelled hollow structure, which is suitable for facilitating the mass transfer and charge transfer.

The HR-TEM images of ZFOs are shown in Fig. 3d–f. The distances between adjacent planes in ZFO-400, ZFO-450, and ZFO-500 represent approximately 0.285, 0.251, and 0.249 nm, respectively, equivalent to the lattice spacing of the (220), (311), and (222) planes for ZnFe_2_O_4_, respectively. The crystal sizes of ZFO-400, ZFO-450, and ZFO-500 are in the order of around 15.6, 15.4, and 17.6 nm, respectively, as described in XRD patterns (Figure S2). For the ZFO-450 of Fig. 3g, the characteristics of the triple-shelled ZnFe_2_O_4_ hollow morphology are separately confirmed by the scanning transmission electron microscopy (STEM) and energy dispersive X-ray analysis (EDX) elemental mappings.

**Figure 4:**
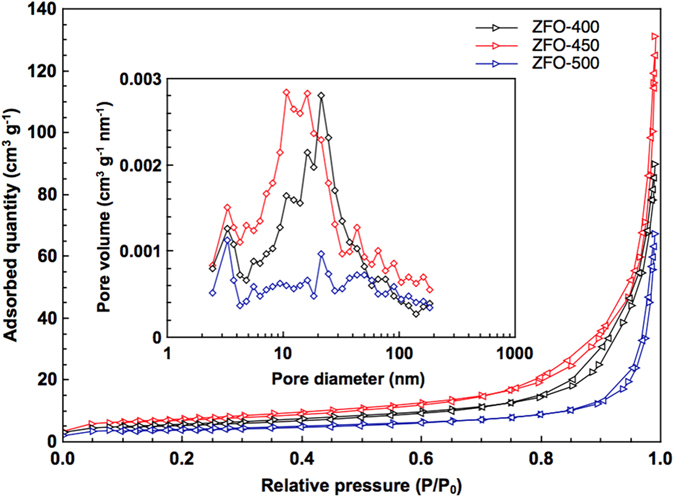
N_2_ adsorption-desorption isotherms and pore size distribution (inset) of ZFO-400, ZFO-450, and ZFO-500.

Figure 4 displays the isotherms of N_2_ adsorption/desorption for ZFO-400, ZFO-450, and ZFO-500. Three isotherms exhibit type IV with H1 hysteresis behaviors caused by the characteristics of mesoporous morphology. The hysteresis loops of ZFO-400 and ZFO-450 are commenced at a relative pressure of 0.7, while that of ZFO-500 starts at a relative pressure of 0.85. Table S1 represents the pore characterization for ZFO-400, ZFO-450, and ZFO-500, which are estimated using BET equation and BJH algorithm. The BET surface area and pore volume for ZFO-450 represent 26.0 m^2^ g^−1^ and 0.183 cm^3^ g^−1^, while those for ZFO-400 and ZFO-500 are 20.1 m^2^ g^−1^ and 0.134 cm^3^ g^−1^, and 14.3 m^2^ g^−1^ and 0.101 cm^3^ g^−1^, respectively. ZFO-450 shows the best pore characteristics due to thin triple-shelled hollow structure as compared to ZFO-400 and ZFO-500 with thick shell hollow structure. From the morphological view, total pore volume and specific surface are adjusted by the calcination temperature, governing the electrochemical performance. The pore size distributions of ZFOs are illustrated in the inset graph of Fig. 4. The pore size distributions of ZFOs are divided into three regions: small mesopore (2–5 nm), large mesopore (5–50 nm), and macropore (>50 nm). The ZFO-450 possesses well-developed pore size distribution and large pore volume forming meso-/macroporous morphology all over the regions as compared to ZFO-400 and ZFO-500. Such meso-/macroporous structures are deeply dependent on interparticle pore spaces within shells and void spaces. ZFO-450 with mesoporous thin triple shells and macroporous wide voids not only suppresses severe volume expansion due to the structural integrity, but also facilitates the electrolyte transfer from the outer shell into the inner shell and void owning to high surface permeability. In addition, the interparticle pore spaces composed of nanoparticles within shells provides more electrochemical active sites for the redox reaction.

**Figure 5:**
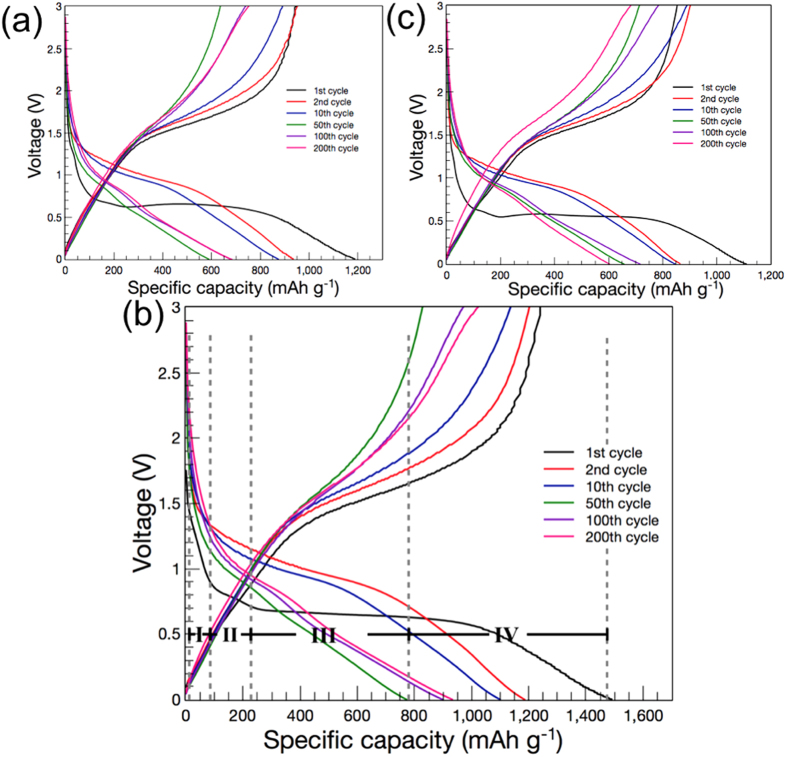
Voltage profiles of (**a**) ZFO-400, (**b**) ZFO-450, and (**c**) ZFO-500 at a current density of 2 A g^−1^ in 1 M LiPF_6_/EC/DMC.

Figure 5a–c shows the discharge (reduction) and charge (oxidation) voltage profiles of ZFOs with different calcination temperatures at a high current density of 2 A g^−1^ between 0.01 V and 3.0 V (vs. Li^+^/Li). The voltage profile of ZFO-450 is much better compared to that of ZFO-400 and ZFO-500. The first charge and discharge capacity of ZFO-450 are approximately 1,102 and 1,412 mA h g^−1^, while those of ZFO-400 and ZFO-500 is around 880 and 1,188 mA h g^−1^, and 853 and 1,120 mA h g^−1^, respectively. The coulombic efficiency at the first cycle are ranked as 78% (ZFO-450) > 76% (ZFO-500) > 74% (ZFO-400), indicating that the irreversibility of ZFO-450 is less than others due to the morphology of triple-shelled microsphere with mesoporous thin shell and macroporous wide void. In the subsequent cycles, the ratios of the capacity retention readily reach to 95, 96, and 99% in the 2^nd^, 10^th^, and 200^th^ cycle, respectively. In the 200^th^ cycle, the discharge capacity for ZFO-450 increase to 932 mA h g^−1^, representing that its morphology is suitable for actualizing better electrochemical performance due to the protection of volume expansion. However, the discharge capacities of ZFO-400 and ZFO-500 at the 200^th^ cycle are much lower than that of ZFO-450, representing 739 mA h g^−1^ and 603 mA h g^−1^. Meantime, while charging and discharging, the behavior of electrochemical performance is mainly governed by the polarization occurring in the range of charging/discharging overpotential. Triple-shelled ZnFe_2_O_4_ hollow microsphere makes it easier to reduce the polarization by overpotential during charging/discharging by facilitating charge transfer and mass transfer due to the formation of thin shell with high mesoporosity and wide void as hollow core with macroporosity. Thus, triple-shelled ZnFe_2_O_4_ hollow microspheres show excellent specific capacity, excellent rate capability, and exceptional cycling stability due to the alleviation of internal resistance and protection of volume expansion^35^,^36^,^37^. As for ZFO-450, the first discharge curve is divided into three regions. It exhibits two obvious voltage plateaus above 0.7 V consisting of a sharp one between 1.5 V and 0.85 V (Region I) and a long one between 0.85 V and 0.7 V (Region II). The discharge capacity in Region I is about 90 mA h g^−1^ indicating that 0.5 lithium ion is inserted (equation 4), while that in Region II is around 225 mA h g^−1^ to be formed Li_0.5_ZnFe_2_O_4_ (equation 5). The long-voltage plateau (Region III) is involved with a consumption of 6.0 mol Li^+^ along with partial reduction of Fe^3+^, Fe^2+^, and Zn^2+^ to Fe^0^ and Zn^0^ as well as the formation of amorphous Li_2_O (equation 6). In region IV between 0.7 V and 0.01 V, the generation of Li-Zn alloy is progressed (equation 7), and the solvent in the electrolyte is irreversibly decomposed to form SEI on the electrode surface. In the subsequent cycles, one broad reduction peak located at 0.9 V as shown in Fig. 5b ascribes to the reversible reduction reaction of amorphous Fe_2_O_3_ and ZnO (equations 8 and 9). On the basis of the mechanism, the Li storage mechanism of ZnFe_2_O_4_ in the first discharge cycle is described as follows^38^:


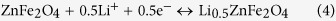



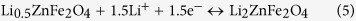



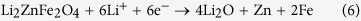



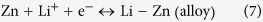


In the following recharging process, the ferrite molecule cannot be recovered and the reactions are progressed as follows:


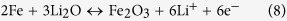



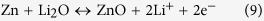


The lithiation and delithiation for ZFO with calcination temperatures (ZFO-400, ZFO-450, and ZFO-500) are estimated by cyclic voltammetry Figure S4. The cyclic voltammograms (CVs) with lithium storage properties were carried out at a scan rate of 0.5 mV s^−1^ in the voltage range of 0.01 to 3 V versus Li/Li^+^. Figure S4 shows the first three CV curves of the ZFO-400, ZFO-450, and ZFO-500, respectively. All patterns of CV curves are nearly analogous. However, the current surface of ZFO-450 is much larger as compared to that of ZFO-400 or ZFO-500, indicating the enhanced electrochemical performance by fast charge transfer. In the first cycle of ZFO-450 (Figure S4b), the ZnFe_2_O_4_ phase is transformed to the Li_0.5_ZnFe_2_O_4_ phase (Region I) in the range of 1.5 to 0.85 V, and then Li_0.5_ZnFe_2_O_4_ phase is transformed to the Li_2_ZnFe_2_O_4_ phase in the range of 0.85 to 0.7 V (Region II). The intense cathodic peak located at ∼0.47 V ascribes to the reduction of Fe^3+^, Fe^2+^, and Zn^2+^ to Fe^0^ and Zn^0^ and subsequent the formation of Li–Zn alloys and Li_2_O. Also, the broad peak situated at ~0.47 V disappears in the following cycles, indicating the irreversible reaction caused by the formation of a solid electrolyte interface (SEI) layer inducing the consumption of excess lithium ion. In the succeeding cycles, one broad reduction peak is observed at 0.9 V, signifying the reversible reduction of amorphous Fe_2_O_3_ and ZnO (equations 8 and 9). During the anodic process, one strong oxidation peak is observed at 1.68 V, which are connected with the oxidation of Zn^0^ and Fe^0^ to Zn^2+^ and Fe^3+^, respectively. In the cathodic process of 2^nd^ and the 3^rd^ cycle, the main peaks shift to about 0.9 V; these shifts are deeply relevant to the easy polarization and the electrochemical reversibility after the initial cycles. The distinctions of main peaks to higher voltage are deeply associated with thin triple-shelled hollow microspheres with extremely mesoporous structure. Two anodic peaks in the third cycle also shift to around 1.73 V representing easy polarization.

**Figure 6:**
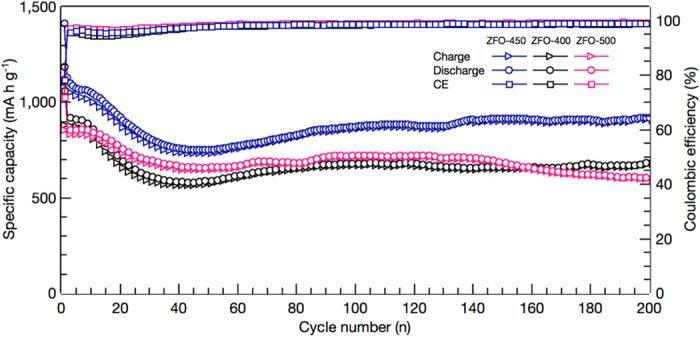
Cycle performances and coulombic efficiencies (CE) of ZFO-400, ZFO-450, and ZFO-500 at a current density of 2 A g^−1^.

The cyclic performances and coulombic efficiencies for ZFOs ranging from 0.01 to 3 V at a high current density 2 A g^−1^ are illustrated in Fig. 6. At the first cycle, the discharge capacities rank as: ZFO-450 (1,412 mA h g^−1^) > ZFO-400 (1,188 mA h g^−1^) > ZFO-500 (1,120 mA h g^−1^), while the coulombic efficiencies rank as: ZFO-450 (78%) > ZFO-500 (76%) > ZFO-400 (74%). The ZFO-450 at the 200^th^ cycle displays the outstanding discharge capacity and exceptional capacity retention. In addition, beginning from excellent discharge capacity of 1184 mA h g^−1^ at the 2^nd^ cycle, it steadily decreases between the 3^rd^ cycle and the 50^th^ cycle, reaches to 909 mA h g^−1^ at the 140^th^ cycle, and then demonstrates the outstanding discharge capacity of 932 mA h g^−1^ at the 200^th^ cycle showing exceptional retention. The specific discharge capacity for ZFO-450 delivers the best value among the ZFOs, and the variance between ZFO-450 and ZFO-400 or ZFO-500 at the 200^th^ cycle surpasses 320 mA h g^−1^ or over. The productive evidences of this electrochemical behavior are originated in the morphology of triple-shelled ZnFe_2_O_4_ hollow microspheres calcined at 450 °C as follows: (i) nano-sized crystallite of 15.4 nm in mean size, (ii) thin outer and inner shells of 50 and 62 nm in mean thickness, (iii) abundant interstitial spaces as the facile pathway of electrolyte due to high surface permeability generated by nanoparticles within shells, (iv) wide void of 1.66 μm in average distance feasible to store the electrolyte that can promote mass transfer, and (v) adequate inner hollow core of 0.95 μm in mean diameter. On the other hand, the specific discharge capacities for ZFO-400 and ZFO-500 representing a similar tendency from the 2^nd^ to the 200^th^ cycle are much lower than that of ZFO-450. The ZFO-400 exhibits the moderate discharge capacity of 934 mA h g^−1^ at the 2^nd^ cycle, sharply decreases to 588 mA h g^−1^ at the 50^th^ cycle, slightly increases to 679 mA h g^−1^ at the 100^th^ cycle, maintains to 658 mA h g^−1^ at the 140^th^ cycle, and then gradually steadily retains 684 mA h g^−1^ at the 200^th^ cycle. Similarly, the ZFO-500 demonstrates 835 mA h g^−1^ at the 2^nd^ cycle, steeply decreases to 651 mA h g^−1^ at the 40^th^ cycle, slightly increases to 708 mA h g^−1^ at the 90^th^ cycle, and then gradually declines to 598 mA h g^−1^ at the 200^th^ cycle. On the other hand, for all of the samples (ZFOs), the discharge capacities decrease in the initial 40 cycles, then increase in the following cycles. This initial capacity loss is ascribed to the formation and conditioning for ZFO electrodes, which is the progress of a stable SEI film formation, ample penetration between the electrode and the electrolyte, and good electronic contact with the current collector. In the following cycling, ZFOs facilitate charge and mass transfer due to mesoporous thin shell and macroporous wide void, leading to the increase in discharge capacity^39^,^40^.

**Figure 7:**
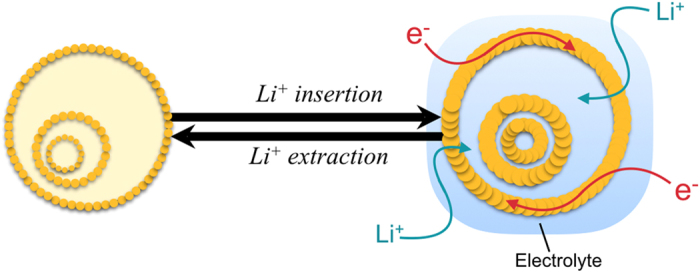
Schematic illustration for the structural variation of triple-shelled hollow microspheres during lithiation/delithiation.

The schematic diagram of the structural variation for triple-shelled hollow spheres in the cycling process is shown in Fig. 7. As illustrated in the structural variation caused by Li^+^ insertion and extraction, thin triple-shelled ZnFe_2_O_4_ hollow microspheres maintain the structural integrity against the severe volume expansion during lithiation/delithiation due to the formation of thin shell by nanoparticles and the guarantee of wide void. More specifically, mesoporous thin shell generated by nanoparticles creates the numerous electrochemical active sites, offers high mass transfer due to the high surface permeability, and reduces the diffusion length enhancing electrochemical kinetics. Thin shells lessen the diffusion time as follows^15^: t = x^2^/2D. Where t indicates the diffusion time, x is the diffusion distance, and D represents the diffusion coefficient. Thus, mesoporous thin shell composed of nanoparticles makes it easier to penetrate the electrolyte into the outer shell as well as the inner hollow core, achieving excellent rate capability by generating numerous electrochemical sites. Meantime, macroporous wide void induces the structural integrity accommodating with the volume expansion occurring during lithiation/delithiation. The wide void and inner hollow core with 1.35 and 0.95 nm in mean space, respectively, act not only as the reservoir of lithium, but also buffer the mechanical stresses by providing the sufficient spaces standing against the severe volume expansion during lithiation/delithiation. The electrochemical performances of different ZnFe_2_O_4_ structures are compared in Table S2^41^,^42^,^43^,^44^,^45^,^46^,^47^.

**Figure 8:**
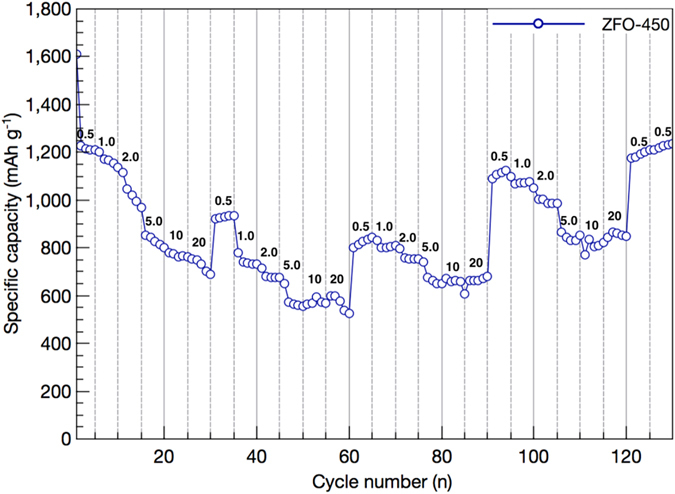
Rate performances of ZnFe_2_O_4_-450 at various current densities from 0.5 to 20 A g^−1^ in 1 M LiPF_6_/EC/DMC.

Figure 8 displays the specific discharge capacity of ZFO-450 with the variation of every 5 cycles at different current densities. At current densities of 0.5, 1, 2, 5, 10, and 20 A g^−1^, the specific capacities were 1228 mA h g^−1^ (2^nd^ cycle), 1200 mA h g^−1^ (6^th^ cycle), 1113 mA h g^−1^ (11^th^ cycle), 853 mA h g^−1^mA h g^−1^ (16^th^ cycle), 780 mA h g^−1^ (21^th^ cycle), and 753 mA h g^−1^ (26^th^ cycle), respectively. At the high current densities of 1, 2, 5, 10, and 20 A g^−1^ on the basis of 0.5 A g^−1^ at the second cycle, the recovery efficiencies reach to 98, 91, 69, 64, and 61%, respectively. During the 120^th^ to the 130^th^ cycle, a current density of 0.5 A g^−1^ is carried out to recheck the recovered specific capacity as compared to the first 5 cycles applied as the exact same amount of current density. The specific capacity sustains 1235 mA h g^−1^ at the 130^th^ cycle, showing outstanding reversibility of the conversion reaction (recovery efficiency: ~100%). There are two reasons for showing superior electrochemical performance of ZFO-450 originated from the triple-shelled hollow structure as follows; (i) the formation of thin shell comprised of nanoparticles creates the electrochemical active sites due to high surface area, facilitates mass transfer due to mesoporous morphology, and easily penetrates the electrolyte from the outer shell into void and inner shell due to high surface permeability, (ii) the wide void serves as the reservoir of electrolyte, provide big space to alleviate the severe volume expansion by fast mass transfer during lithiation/delithiation, and plays an important in maintaining the structural integrity accommodating mechanical strains by volume expansion^48^,^49^.

Figure S5 illustrates the electrochemical impedance spectroscopy (EIS) diagrams offering the information of the structural stabilities in the electrochemical process for ZFOs calcined at 400, 450, and 500 °C. The results of impedance measurements are conducted at room temperature in a wide frequency range from 100 kHz to 10 mHz for a sample before cycling and a sample after 100 cycling at a current density of 2 A g^−1^. All of the plots are composed of each semicircle in the high-middle frequency regions and an inclined line at low frequencies^24^. The charge-transfer resistance (R_ct_) at a middle frequency semicircle represents the interfacial impedance between electrode and electrode, and the lithium diffusion transfer. For fresh ZFOs (before cycling) in Figure S5a, the charge transfer resistance of the ZFO-450 is much higher compared to those of ZFO-400 and ZFO-500. The rise in charge transfer resistance is originated from the insufficient contact between the active material and electrolyte because the electrolyte is not sufficiently penetrated into the shells and voids of ZFO-450. As for ZFOs after 100 cycling in Figure S5b, the charge transfer resistance of ZFO-450 with thin triple-shelled hollow microsphere is decreased due to the improvement of the charge transfer process over the electrode surface, which is associated with abundant interparticle pore spaces and sufficient void spaces with larger specific surface area. The morphology of thin triple-shelled ZnFe_2_O_4_ hollow microsphere is well suitable for the remarkable improvement of the cycling capacity and rate capability.

## Methods

### Thin triple-shelled ZnFe_2_O_4_ hollow microspheres

Thin triple-shelled ZnFe_2_O_4_ hollow microspheres (ZFO) were prepared by a one-pot hydrothermal technique and subsequent calcination process in air. 0.83 mmol of zinc nitrate (Zn(NO_3_)_2_·6H_2_O), 1.67 mmol of iron nitrate (Fe(NO_3_)_2_·9H_2_O), and 25 mmol of sucrose (C_12_H_22_O_11_) were dissolved in 50 ml of H_2_O and 50 ml of ethylene glycol (EG: CH_2_OH)_2_. EG serves as dispersing reagent of metal precursors in a hydrothermal process. The resulting solution was then moved into 180 ml of poly(tetrafluoroethylene) (PTFE)-lined stainless steel autoclave. After that, the sealed autoclave was reacted at 190 °C for 24 h, and then cooled down to room temperature in the autoclave. The obtained products were washed using distilled water several times. Finally, ZFOs were synthesized by calcining as-prepared precursors at 400, 450, and 500 °C for 4 h at a heating rate of 2 °C min^−1^ in air. The obtained ZnFe_2_O_4_ hollow microspheres were denoted as ZFO-400, ZFO-450, and ZFO-500 with different calcination temperatures.

### Materials characterizations

The surface morphologies of ZFOs were analyzed by the field emission scanning electron microscopy (FE-SEM, S-4700, Hitachi Japan). The nanostructured morphology and elemental mappings of ZFOs were scrutinized by the transmission electron microscopy (TEM, TECNAI F20, Philips, Netherlands) in the Korean Basic Science Institute (KBSI, Gwangju Center). The determination of crystallinity was investigated by the X-ray diffraction (XRD, D/MAX Ultima III, Rigaku, JAPAN). The thermal characterization of ZFOs was illustrated by thermogravimetric analysis (TGA, Shimadzu, TA-50, JAPAN) at a ramping rate of 5 °C min^−1^ under air atmosphere. The porous characteristics of the samples were verified at 77 K in nitrogen atmosphere by the Brunauer-Emmett-Teller (BET, Micromeritics ASAP2020, USA) method.

### Electrochemical characterizations

The electrochemical performances were carried out using coin cells (type CR2032). The slurry was made by completely mixing 70 wt.% as-prepared active materials, 20 wt.% carbon black (Super P), and 10 wt.% poly(acrylic acid) (PAA, Mw: 450,000, Aldrich) in N-methyl pyrrolidinone (NMP). The working electrodes were prepared by casting the slurry on the surface of copper foil and then dried at 120 °C for 12 h in a vacuum oven. The mass loading of ZFO on copper foil was accurately controlled around 0.80 mg cm^−2^. The coin cell was assembled in an Ar-filled glove box. Lithium foil was used as a counter and a reference electrode. The electrodes were separated by polypropylene films (Celgard^®^ 2400). The electrolyte was composed of a solution of 1 M LiPF_6_ in a mixture of ethylene carbonate (EC) and dimethyl carbonate (DMC) (1:1, v/v). The specific charge and discharge performance of the ZFOs were carried out by using a battery cycler (WBCS 3000, Won-A Tech., Korea) with different current densities (0.5, 1, 2, 5, 10, and 20 A g^−1^). The measurements by cycling voltammetry (CV) were carried out using an electrochemistry workstation (IM6e, Zahner Electrik, Germany) ranging from 0.01 to 3.0 V at a scan rate of 0.5 mV s^−1^. The Nyquist plots as the results of electrochemical impedance spectroscopy (EIS) were expressed graphically by using an electrochemistry workstation (IM6e, Zahner Electrik, Germany) in the range of 10 mHz to 100 kHz.

## Discussion

We have successfully designed a noble architecture of thin triple-shelled ZnFe_2_O_4_ hollow microspheres (ZFOs) with mesoporous and macroporous morphology for lithium ion battery anodes with different calcination temperatures. The ZFOs is synthesized using ZnFe-glycolate/carbon microspheres obtained by a one-pot hydrothermal reaction, and decomposition of core carbon at an optimum calcination temperature. The preparation of ZFOs are mainly governed by a mutual cooperation of inward and outward ripening with different calcination temperatures, influencing on the formation of thin shells with mesoporous structure and wide void spaces with macroporous structure. The ZFO-450 provides superior electrochemical performance involving excellent rate capability and exceptional cycling retention, maintaining at 932 mA h g^−1^ at a high current density without any capacity fading at the 200^th^ cycle. This rate capability is ascribed to thin triple-shelled hollow structure with well-developed mesoporous/macroporous structure. The ZFO with thin shells and wide voids creating a highly porous morphology offers a large number of electrochemically active sites, facilitates Li^+^ insertion/extraction inducing the improvement of rate capability and cycling stability. Concurrently, the wide void in thin ZFO-450 makes it easier to alleviate severe volume expansion standing against fast lithiation/delithiation without any pulverization.

## Additional Information

**How to cite this article**: Hwang, H. *et al*. Effects of calcination temperature for rate capability of triple-shelled ZnFe_2_O_4_ hollow microspheres for lithium ion battery anodes. *Sci. Rep.* **7**, 46378; doi: 10.1038/srep46378 (2017).

**Publisher's note:** Springer Nature remains neutral with regard to jurisdictional claims in published maps and institutional affiliations.

## Supplementary Material

Supplementary Information
