# Supplementary material for: Effects of calcination temperature for rate capability of triple-shelled ZnFe2O4 hollow microspheres for lithium ion battery anodes
Source: Sci Rep. 2017 Apr 18;7:46378. doi: 10.1038/srep46378 (PMC5394453; doi:10.1038/srep46378)

**Supplementary information**

**Effects of calcination temperature for rate capability of triple-shelled ZnFe_2_O_4_ hollow microspheres for lithium ion battery anodes**

Hojin Hwang, Haeun Shin, and Wan-Jin Lee*

School of Chemical Engineering, Chonnam National University,
Gwangju 61186, Republic of Korea
*E-mail: wjlee@jnu.ac.kr


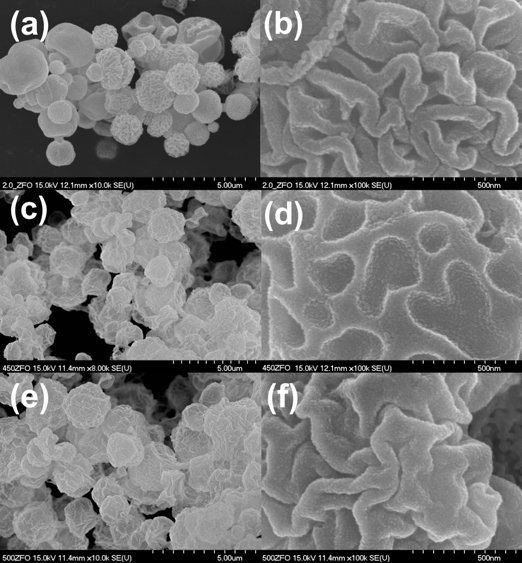


**Figure S1.** SEM images for (a,b) ZFO-400, (c,d) ZFO-450, and (e,f) ZFO-500 in air.


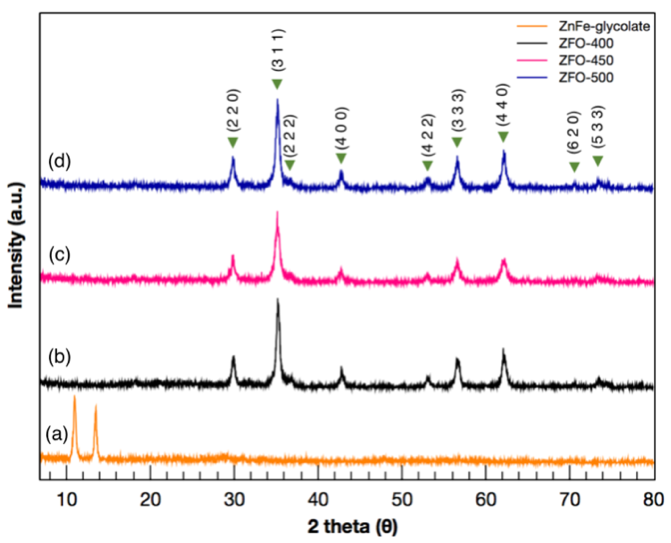


**Figure S2.** XRD patterns of (a) Zn-Fe/glycolate, (b) ZFO-400, (c) ZFO-450, and (d) ZFO-500.


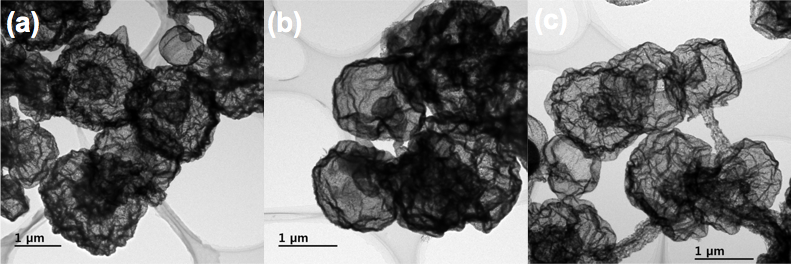


**Figure S3.** HRTEM images for (a) ZFO-400, (b) ZFO-450, and (c) ZFO-500.


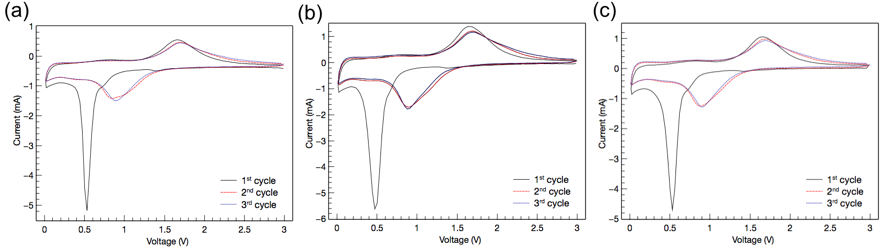


**Figure S4.** Cyclic voltammograms of (a) ZFO-400, (b) ZFO-450, and (c) ZFO-500 at a scanning rate of 0.5 mV s^-1^ in 1 M LiPF_6_/EC/DMC electrolyte.

**
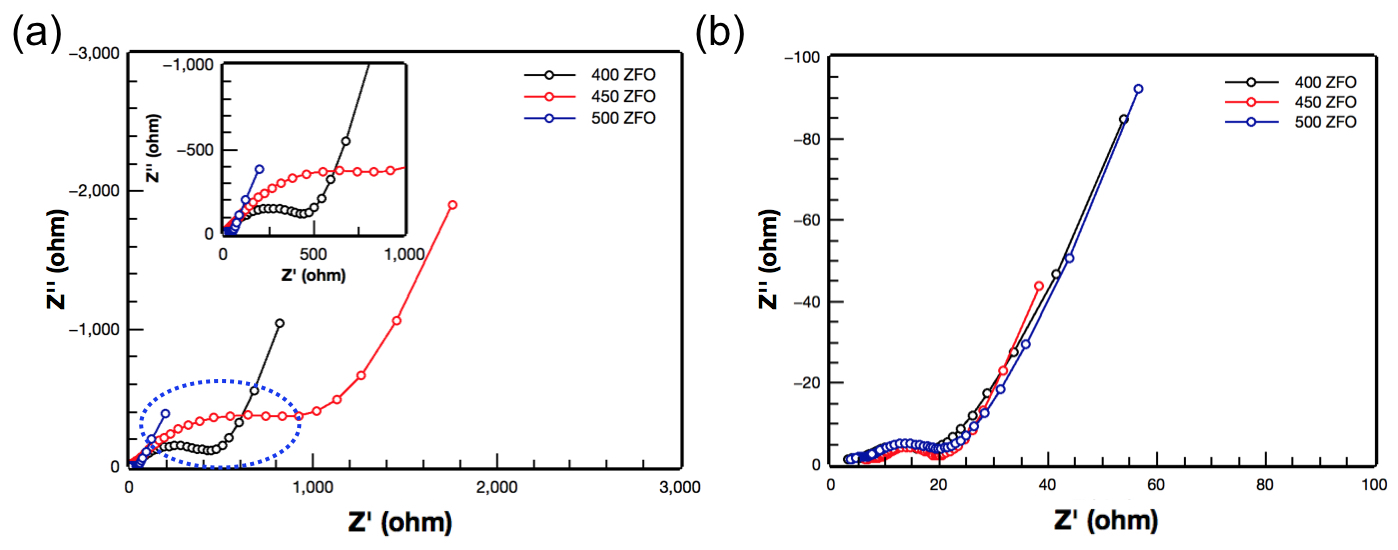
**

**Figure S5.** AC impedance spectra of ZFO-400, ZFO-450, and ZFO-500 (a) before and (b) after 100 cycles.

**
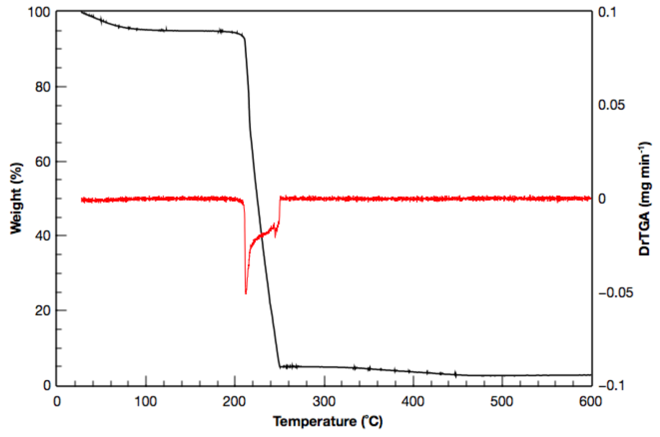
**

**Figure S6.** TGA and DTA curves of ZnFe-glycolate/carbon microspheres.

**Table S1.** Pore characteristics of ZFO-400, ZFO-450, and ZFO-500


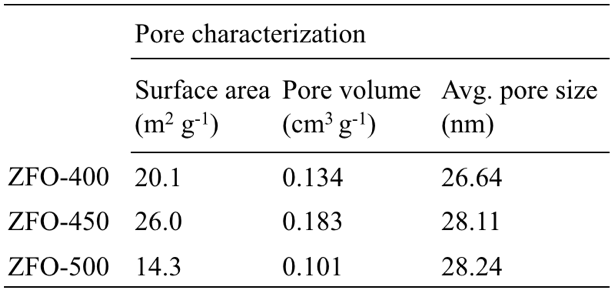


**Table S2.** Comparison of electrochemical performances for different ZnFe_2_O_4_ electrodes


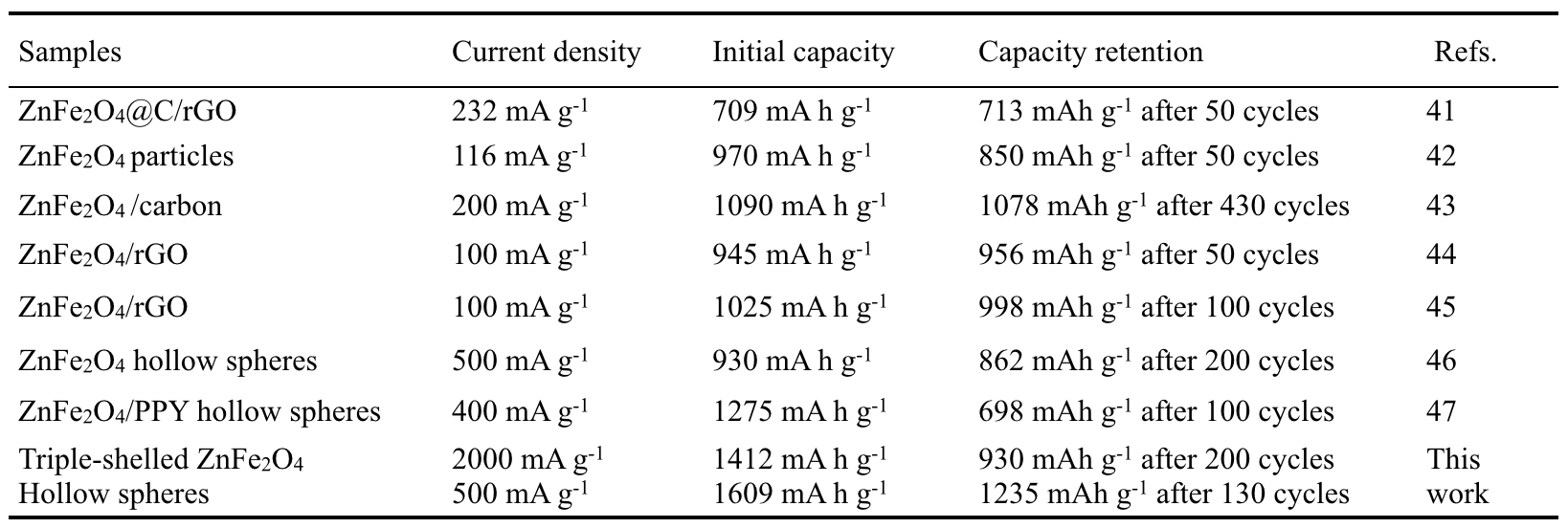

Supplement: Supplementary Information [file srep46378-s1.docx]
